# Homogeneously high expression of CD32b makes it a potential target for CAR-T therapy for chronic lymphocytic leukemia

**DOI:** 10.1186/s13045-021-01160-9

**Published:** 2021-09-16

**Authors:** Guoling Wang, Xiaolei Sun, Shiyu Zuo, Chuo Li, Qing Niu, Yonghui Xia, Yuan Meng, Min Liu, Zihao Fang, Xi Yang, Yanyu Jiang, Sheng Wang, Haidong Cui, Huifang Huang, Erlie Jiang, Dongming Zhou, Qi Deng, Jing Pan, Xiaoming Feng

**Affiliations:** 1 State Key Laboratory of Experimental Hematology, National Clinical Research Center for Blood Diseases, Institute of Hematology and Blood Diseases Hospital, Chinese Academy of Medical Sciences and Peking Union Medical College, Tianjin, 300020 China; 2 Key Laboratory of Immune Microenvironment and Disease of the Ministry of Education, Tianjin Medical University, Tianjin, 300070 China; 3 Institut Pasteur of Shanghai, Chinese Academy of Sciences, Shanghai, 200031 China; 4 Department of Hematology, Tianjin First Central Hospital, School of Medicine, Nankai University, Tianjin, 300192 China; 5 Department of Thoracic Surgery, Hubei Cancer Hospital, Tongji Medical College of Huazhong University of Science and Technology, Wuhan, 430079 China; 6 Department of Breast Surgery, The First Affiliated Hospital, School of Medicine, Zhejiang University, Hangzhou, 310000 China; 7 Central Laboratory, Fujian Medical University Union Hospital, Fuzhou, 350001 China; 8 Department of Pathogen Biology, School of Basic Medical Sciences, Tianjin Medical University, Tianjin, 300070 China; 9 State Key Laboratory of Experimental Hematology, Boren Clinical Translational Center, Department of Hematology, Beijing Boren Hospital, Beijing, 100070 China

**Keywords:** Chronic lymphocytic leukemia, CD32b, Chimeric antigen receptor, Antigen site density

## Abstract

CD19 chimeric antigen receptor (CAR)-T cells have been used to treat patients with refractory chronic lymphocytic leukemia (CLL). However, approximately 50% of patients do not respond to this therapy. To improve the clinical outcome of these patients, it is necessary to develop strategies with other optimal targets to enable secondary or combinational CAR-T cell therapy. By screening a panel of surface antigens, we found that CD32b (FcγRIIb) was homogeneously expressed at high site density on tumor cells from CLL patients. We then developed a second-generation CAR construct targeting CD32b, and T cells transduced with the CD32 CAR efficiently eliminated the CD32b^+^ Raji leukemic cell line in vitro and in a mouse xenograft model. Furthermore, CD32b CAR-T cells showed cytotoxicity against primary human CLL cells that were cultured in vitro or transplanted into immunodeficient mice. The efficacy of CD32b CAR T cells correlated with the CD32b density on CLL cells. CD32b is not significantly expressed by non-B hematopoietic cells. Our study thus identifies CD32b as a potential target of CAR-T cell therapy for CLL, although further modification of the CAR construct with a safety mechanism may be required to minimize off-target toxicity.

**Supplementary Information:**

The online version contains supplementary material available at 10.1186/s13045-021-01160-9.

**To the editor:**

Chronic lymphocytic leukemia (CLL) is a hematological neoplasm mostly diagnosed in the elderly. Refractory and relapsed (r/r) CLL patients have a poor prognosis with limited therapeutic options [1, 2]. Chimeric antigen receptor (CAR)-T cells targeting CD19 have shown activity in CLL, but can only induce complete remission in about 30%-60% of the patients [3, 4]. It is essential to develop alternative targets for secondary or combinational CAR-T cell therapies for CLL.

Since target antigen site density and expression percentage on tumor cells are critical determinants of CAR-T cell efficacy [5], we aimed to identify a target antigen that was expressed at high levels on all CLL cells. The expression levels of B cell-associated antigens (CD19/CD20/CD22/CD32) and 3 previously suggested targets (CD23/ROR1/FcμR) were examined on leukemic cells from CLL patients (Additional file 1: Table S1). CD32 (FCGR2) was expressed on 100% CD5^+^CD19^+^ CLL cells from all patients, similar to CD19 (Fig. 1a, Additional file 3: Fig. S1a). The average site density of CD32 was much higher than that of CD19 and the other antigens tested (Fig. 1b, Additional file 3: Fig. S1b). CD32 has three isoforms, CD32a, b, c; CD32b shares the same extracellular domain with CD32c [6]. RNA sequencing revealed that leukemic cells and Raji cells expressed abundant CD32b and low levels of CD32c but little CD32a (Fig. 1c). A soluble scFv derived from the CD32b-specific antibody 2B6 confirmed the homogeneously high expression of CD32b on CLL (Figs. 1d–f, Additional file 3: Fig. S1d). CD32b was not significantly expressed on hematopoietic stem/progenitor cells and most mature blood cells, but was expressed in a small proportion of dendritic cells (Fig. 1g–h).

**Fig. 1:**
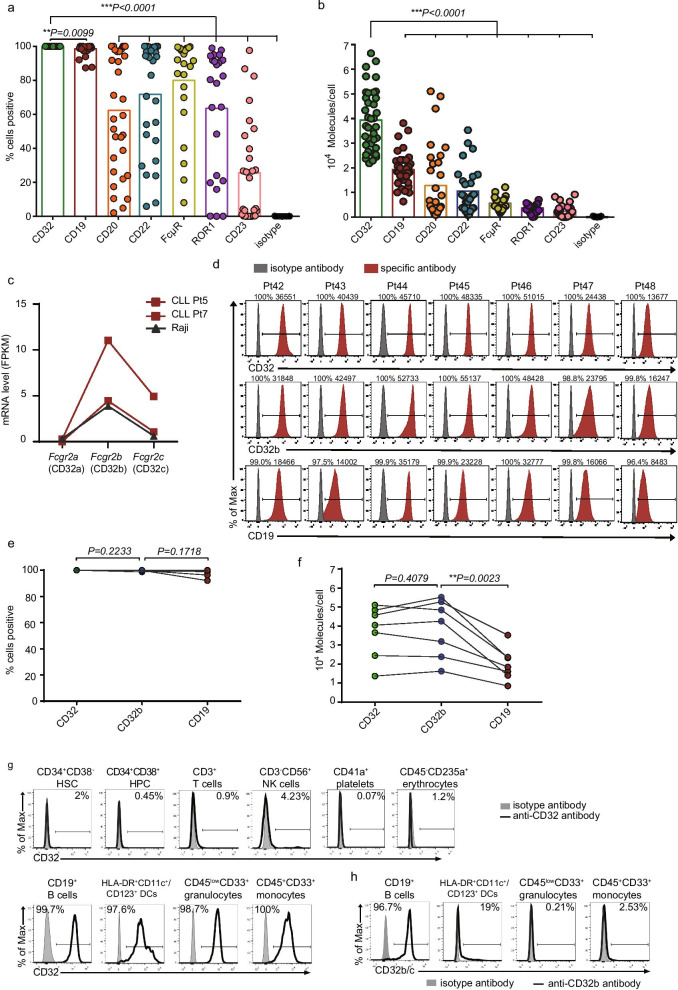
CD32b is homogeneously expressed at high level on primary CLL cells, but not significantly expressed on non-B hematopoietic cells. **a** Expression (% positive) of CD32 (n = 41), CD19 (n = 41), CD20 (n = 33), CD22 (n = 29), CD23 (n = 29), ROR1 (n = 22) and FcμR (n = 22) in CLL samples (from CLL patients in Additional file 1: Table S1). **b** Evaluation for site density of CD32 and other antigens in CLL patients (sample size was the same as **a**) using Quantibrite-PE beads. **c** Transcriptional profile of Fcgr2a, Fcgr2b and Fcgr2c from 2 CLL samples and Raji cell line by RNA sequencing. **d** Flow cytometric analysis of surface expression of CD32, CD32b and CD19 in 7 CLL patients. **e** Expression (% positive) of CD32, CD32b and CD19 in CLL patients (n = 7). **f** Site density comparison among CD32b, CD32 and CD19 in CLL patients (n = 7). Data in **e**–**f** belong to Pt 42–48, and the expression of CD32 and CD19 on samples from Pt 42–48 is not included in **a**–**b**. **h** Flow cytometric analysis of surface expression of CD32 on peripheral blood cells and HSPCs (CD34^+^ CD38^−^ HSCs and CD34^+^ CD38^+^ HPCs) from a healthy donor. **i** Flow cytometric analysis of CD32b expression on normal peripheral blood cells and HSPCs from a healthy donor. FPKM: expected number of Fragments Per Kilobase of transcript sequence per Millions base pairs sequenced. HSC, hematopoietic stem cell; HPC, hematopoietic progenitor cell; NK, natural killer; DC, dendritic cells. Data were representative of two independent experiments. Unpaired two-tailed Student's t test was used for statistical analyses in **a**, **b**; paired two-tailed Student's t test was used in **e** and **f** (*P < 0.05, **P < 0.01, ***P < 0.001)

Second-generation CAR constructs with scFv derived from the CD32b-specific antibodies 2B6 and NOV2108 were developed (Fig. 2a, b, Additional file 2). Since the CLL cell line MEC1 only partially expressed CD32, we used the Raji cell line, which had homogeneous CD32b expression, to evaluate the activity of CD32b CAR-T cells (Additional file 3: Fig. S2a, b). 2B6bbz showed slightly higher cytotoxicity against Raji cells than did 2108bbz in vitro, and 2B6bbz T cells proliferated and strongly diminished the leukemia burden and prolonged survival in Raji-engrafted mice (Fig. 2c-i, Additional file 3: Fig. S2c, d).

**Fig. 2:**
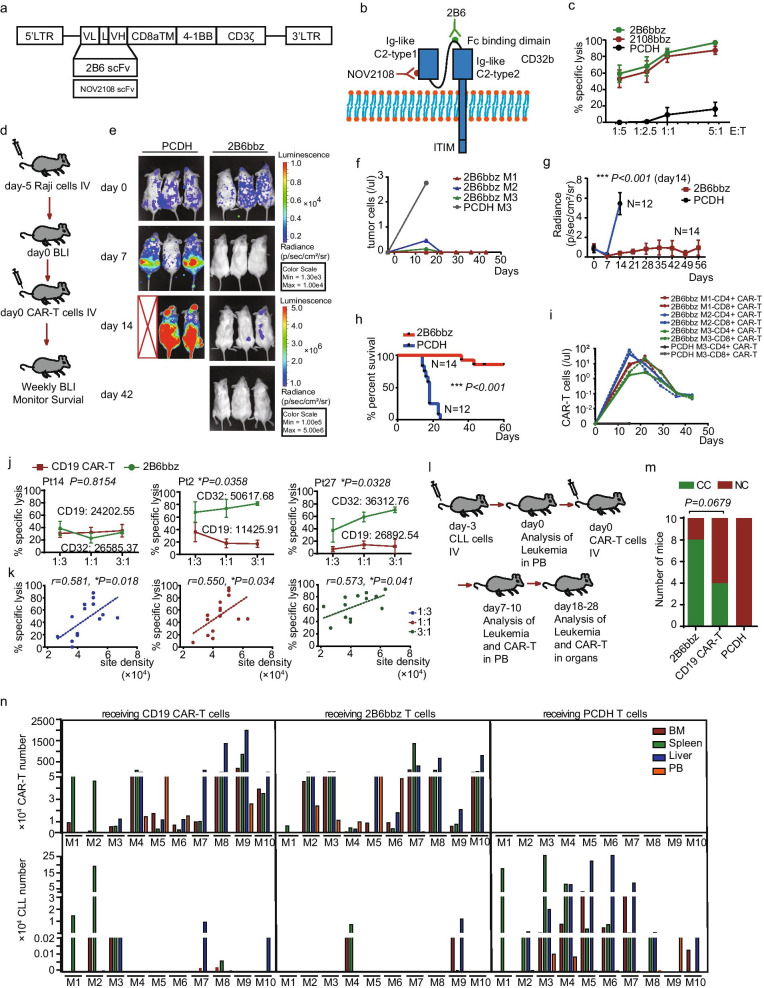
CD32b CAR-T efficacy against Raji cells and primary CLL cells. **a** Diagram indicating constructions of two CD32b CAR sequences (scFvs from clone 2B6 or NOV2108). **b** NOV2018 scFv binds Ig-like C2-type 1 domain of CD32b, whereas 2B6 binds binding domain of CD32b. **c** Cytotoxicity of CD32b CAR-T targeting Raji cells after incubation for 36 h at the indicated effector-to-target (E: T) ratios; control T cells were used as negative controls. **d** Schematic of the Raji xenograft model. NSG mice were injected via tail vein with 3 × 10^5^ luciferase^+^ Raji cells on day-5. Bioluminescent imaging was performed on day 0 to quantify engraftment and then weekly measured. Control T cells or 2B6bbz T cells (1 × 10^6^) were injected IV on day 0. **e** Representative bioluminescent imaging at day 0, 7, 14 and 42 after injection of Raji cells. **f** Flow cytometric analysis of Raji cells in peripheral blood from Raji-NSG mice (from e). **g** Bioluminescent signal for each treatment group over time. Data represent mean values of each group ± SD. **h** Log-rank survival curve was used for survival analysis of Raji xenograft mice treated by 2B6bbz or control T cells. Data of g and h were summarized from 4 independent experiments. (Control, n = 12; 2B6bbz, n = 14). **i** Flow cytometric analysis of CAR-T cells in peripheral blood from Raji-NSG mice (from e). **j** Specific cytotoxicity targeting of CLL by 2B6bbz and CD19 CAR-T cells after incubation with primary CLL cells for 36 h at the indicated E:T ratios; Three representative CLL patient examples are shown. **k** Correlation between 2B6bbz T cytotoxicity and CD32 density across different patient CLL samples. **l** Schematic of the primary CLL xenograft model. NSG mice were sublethally irradiated (150 cGy) on day -3 and injected with 2–4 × 10^7^ CLL PBMCs via the tail vein on day -3. Engraftment was confirmed by flow cytometry in PB around day 0. Mice were then injected with 5 × 10^5^ 2B6bbz T, CD19 CAR-T cells or control T cells via the tail vein and bled weekly to quantify CLL burden. **m** Response of primary CLL-NSG mice treated with 2B6bbz T (CC, n = 8; NC, n = 2), CD19 CAR-T (CC, n = 4; NC, n = 6) or control T cells (NC, n = 10). **n** Number of CAR-T and tumor residue in PB, BM, liver and spleen from CLL-NSG mice after receiving CAR-T cells for 18 days. Data of m and n were summarized from four independent experiments. M indicates mouse. CC, complete clearance (defined as tumor residual less than 0.001% in all the tissues detected); NC, not clearance (mouse couldn’t be defined as CC); BM, bone marrow; PB, peripheral blood. Chi-square test was used for statistical analysis in m. Log-rank (Mantel–Cox) test was used for statistical analysis in h. Unpaired two-tailed Student's t test was used for statistical analyses in g and j. Pearson correlation analysis was used in k. (*P < 0.05, **P < 0.01, ***P < 0.001)

In vitro cytotoxicity of 2B6bbz to primary CLL cells was higher than that of 2108bbz (Additional file 3: Fig. S3b). 2B6bbz T cells displayed similar anti-CLL cytotoxicity with CD19 CAR-T cells when the expression of CD19 and CD32 in leukemia was similar, and 2B6bbz was superior to CD19 CAR-T cells when the expression of CD32 in leukemia was higher than CD19 (Fig. 2j). Moreover, cytotoxicity of 2B6bbz T cells positively correlated with CD32 density across different samples (Fig. 2k).

The in vivo anti-CLL activity of 2B6bbz T cells was assessed in NSG mice transplanted with patient samples (Fig. 2l). 2B6bbz T cells were as potent as CD19 CAR-T cells: they achieved complete clearance of CLL in 80% (8/10) of mice and showed robust proliferation in most mice (Fig. 2m-n, Additional file 3: Fig. S4b, c). Loss of CD32b expression was not observed (Additional file 3: Fig. S4d). Due to the limited persistence of CLL in mice, we could not evaluate whether 2B6bbz T cells could provide a long-term cure effect. Since this model has been widely used to evaluate the in vivo efficacy of new drugs in CLL [7], our results indicate that CD32b CAR-T cells have potent cytotoxicity against CLL cells in vivo.

CD32b CAR-T cells may cause B cell aplasia, which can be managed with immunoglobulin infusion. Previous reports have shown the expression of CD32b in some normal tissues and cells, including airway smooth muscle cells, liver sinusoidal endothelial cells, Kupffer cells and placenta [8, 9], which may cause potential off-target toxicities of CD32b CAR-T cell therapy. However, CD32b may still be an applicable target, since the potential off-target toxicity could be alleviated by decreasing CAR affinity for antigen or adopting a synNotch or zipper safety gate, which has been validated in various CAR-T cell studies [10–12]. Therefore, it would be feasible to improve the safety of CD32b CAR-T cells based on these modifications.

In summary, our study identifies CD32b as an antigen that is homogeneously expressed at high levels on CLL cells. CD32b CAR-T cells showed killing efficacy against primary CLL cells in vitro and in vivo. CD32b is therefore a promising target for CAR therapy in CLL, although further evaluation of off-target toxicities and optimization with safety modifications are needed before conducting clinical trials.

## Supplementary Information

**Additional file 1: Table S1.** Patients’ information and expressional characteristics of all antigens.

**Additional file 2.** Materials and Methods.

**Additional file 3: Fig. S1.** CD32 expresses higher than other antigens on primary CLL samples. **a** Flow cytometric analysis of surface expression of CD32, CD19, CD20, CD22, ROR1, FcμR and CD23 in 4 CLL samples. CLL cells were gated as CD19^+^CD5^+^ cells. **b** Site density comparison between CD32 and CD19 in CLL patients (n = 41). **c** Quantification of mRNA transcripts from Raji cells, THP-1 cells and leukemic cells of 2 CLL patients by RNA sequencing. **d** 293T cells were genetically modified to express CD32a and CD32b; cells (unmodified 293T, CD32a^+^ 293T and CD32b^+^ 293T) were stained with 2B6-scFv-Flag Ab, 2108-scFv-Flag Ab and anti-CD32 mAb (clone FUN-2). **Fig. S2.** CD32b CAR-T against Raji cells in vitro and in vivo. **a** Flow cytometry analysis of CAR expression in T cells following lentiviral transduction. Left, control T cells; middle, T cells transduced with 2B6bbz; right, T cells transduced with 2108bbz. CARs were detected by CD32b-His followed by an anti-His-APC second antibody stain. **b** Flow cytometric analysis of surface expression of CD32b on the B-cell leukemia cell lines Mec-1 and Raji. **c** Antigen-specific cytokine production in response to CD32b^+^ Raji cells. 2B6bbz and control T cells were incubated with Raji cells (2 × 10^4^) respectively for 24 h in E: T ratio of 1:1. The various proteins in the culture supernatant were detected using the bead-based “LEGENDplex multi-analyte assay.” **d** Representative flow cytometric plot and flow gating strategy of peripheral blood from Raji-NSG mice 15 days after receiving 2B6bbz or control T cells. **Fig. S3.** CD32b CAR-T had potent cytotoxicity to primary CLL. **a** Flow cytometry analysis of CAR expression in T cells following lentiviral transduction. Left, control T cells; left-center, T cells transduced with 2B6bbz; right-center, T cells transduced with 2108bbz; right, T cells transduced with CD19 CAR. CARs were detected by CD32b-His/CD19-Fc followed by an anti-His-APC/anti-Fc second antibody stain. **b** Specific cytotoxicity of 2B6bbz, 2108bbz or control T cells after coculture with primary CLL cells for 36 h at the indicated E:T ratios; **c** Antigen-specific cytokine production of 2B6bbz, CD19 CAR-T and control T cells in response to 24 h co-culturing with primary CLL cells. **Fig. S4.** 2B6bbz T against primary CLL cells in vivo. **a** Flow cytometric analysis of tumor percentage in peripheral blood before T cells infusion. **b** Representative flow cytometry plot of bone marrow, spleen and peripheral blood, from CLL-NSG mice after receiving CAR-T cells for 18 days. **c** Treatment response of 2B6bbz and CD19 CAR-T cells against mice transplanted with different antigen density primary CLL cells. **d** Flow cytometric analysis of CD32 and CD19 expression on CLL cells from peripheral blood of NSG mice before and at 7 days after CAR-T infusion. **e** Quantification of the percentage of CLL cells in peripheral blood, bone marrow, spleen and liver from CC and NC CLL-NSG mice after receiving CAR-T cells for 18 days. **f** Number of CAR-T cells in peripheral blood, bone marrow, spleen and liver from CC and NC CLL-NSG mice after receiving CAR-T cells for 18 days. **Fig. S5.** mRNA expression profile of *fcgr2b* and *fcgr2c* in normal human tissues at mRNA level according to publicly available database (BioGPS: *fcgr2b*, http://biogps.org/#goto=genereport&id=2213; *fcgr2c*, http://biogps.org/#goto=genereport&id=9103)

## Data Availability

All data needed to evaluate the conclusions in the paper are present in the paper or the additional files.

## Abbreviations

CAR: Chimeric antigen receptor
CLL: Chronic lymphocytic leukemia
CR: Complete response
r/r: Refractory and relapsed
FcγRIIb: Low affinity immunoglobulin gamma Fc region receptor II-b
mAb: Monoclonal antibodies
scFv: Single-chain variable fragment
FPKM: Expected number of Fragments Per Kilobase of transcript sequence per Millions base pairs sequenced
PBMC: Peripheral blood mononuclear cell
PB: Peripheral blood
CC: Complete clearance
NC: Not clearance
HSC: Hematopoietic stem cell
HPC: Hematopoietic progenitor cell
BM: Bone marrow
NK: Natural killer
DC: Dendritic cells
