# Supplementary material for: Homogeneously high expression of CD32b makes it a potential target for CAR-T therapy for chronic lymphocytic leukemia
Source: J Hematol Oncol. 2021 Sep 16;14:149. doi: 10.1186/s13045-021-01160-9 (PMC8447616; doi:10.1186/s13045-021-01160-9)
Supplement: Supplementary file 1 — Additional file 1: Table S1. Patients’ information and expressional characteristics of all antigens. [file 13045_2021_1160_MOESM1_ESM.pdf]

Supplementary Table 1

| | | | | postive rate (%) | | | | | | | |
|---|---|---|---|---|---|---|---|---|---|---|---|
| patient no. | gender | age | diagnosis | CD32 | CD19 | CD20 | CD22 | CD23 | ROR1 | FcμR | CD32B |
| 1 | female | 55 | CLL | 100 | 99.8 | 51 | 50.1 | 47.5 | 77.3 | 69.9 | |
| 2 | male | 54 | CLL | 100 | 97.7 | 47.8 | 48.1 | 97.6 | 91.5 | 99 | |
| 3 | female | 52 | CLL | 100 | 100 | 99.9 | 100 | 0.14 | 0.019 | 94.6 | |
| 4 | male | 61 | CLL | 100 | 89.9 | 6.41 | 6.43 | 1.73 | 94.4 | 13.8 | |
| 5 | female | 64 | CLL | 100 | 97.5 | 10.1 | 32.6 | 0.036 | 91.9 | 40.3 | |
| 6 | male | 53 | CLL | 100 | 94 | 99.9 | 100 | 22.7 | | | |
| 7 | male | 70 | CLL | 100 | 100 | 22 | 91.5 | 39.7 | | | |
| 8 | male | 55 | CLL | 100 | 100 | 34.4 | 93.5 | 51.6 | | | |
| 9 | female | 72 | CLL | 100 | 100 | 26.2 | 83 | 26.7 | | | |
| 10 | male | 68 | CLL | 100 | 99.8 | 2.09 | 24.3 | 3.36 | | | |
| 11 | male | 70 | CLL | 100 | 99.7 | 17.5 | 67.9 | 27.3 | 97.8 | 92.1 | |
| 12 | male | 73 | CLL | 100 | 99.9 | 100 | 100 | 4.22 | 0.088 | 98.8 | |
| 13 | male | 49 | CLL/SLL | 100 | 100 | 51.3 | 54 | 34.1 | 80.4 | 91.6 | |
| 14 | male | 71 | CLL | 100 | 99.8 | 100 | 100 | 0.21 | 20.9 | 84 | |
| 15 | male | 86 | CLL | 100 | 99.9 | 94.3 | 99.6 | 4.59 | 99 | 98.9 | |
| 16 | male | 74 | CLL | 100 | 99.9 | 84.9 | 55.3 | 82.5 | 95.1 | 97.6 | |
| 17 | male | 61 | CLL | 100 | 100 | 69 | 96.8 | 26.1 | 90.1 | 97.1 | |
| 18 | male | 55 | CLL | 100 | 99.9 | 91.8 | 94.5 | 86.8 | 19.8 | 88.2 | |
| 19 | male | 75 | CLL | 100 | 99.8 | 57.3 | 7.92 | 26.7 | 91.8 | 95.5 | |
| 20 | male | 53 | CLL（Richer） | 100 | 99.8 | 24 | 23.9 | 3.5 | 78.3 | 21.4 | |
| 21 | male | 64 | CLL | 100 | 99.8 | 46.8 | 29.6 | 56.3 | 82.6 | 83.4 | |
| 22 | male | 57 | CLL | 100 | 98.9 | 100 | 99.6 | 0.24 | 0.14 | 61.2 | |
| 23 | male | 68 | CLL | 100 | 100 | | | | 91.1 | 97.5 | |
| 24 | male | 72 | CLL/SLL | 100 | 99.9 | | | | 48.4 | 98.9 | |
| 25 | female | 63 | CLL | 100 | 99.9 | | | | 64.3 | 99.3 | |
| 26 | male | 68 | CLL | 100 | 99.8 | | | | 94.4 | 76.2 | |
| 27 | male | 65 | CLL | 100 | 98.5 | | | | 95 | 99.6 | |
| 28 | female | 76 | CLL | 100 | 100 | 71.2 | | | | | |
| 29 | male | 68 | CLL | 100 | 99.1 | 10.5 | | | | | |
| 30 | male | 73 | CLL | 99.9 | 87.7 | 98.6 | | | | | |
| 31 | male | 71 | CLL | 100 | 100 | 59.4 | | | | | |
| 32 | male | 58 | CLL | 100 | 100 | | | | | | |
| 33 | female | 53 | CLL | 100 | 100 | | | | | | |
| 34 | male | 66 | CLL | 100 | 94.8 | | | | | | |
| 35 | male | 54 | CLL | 100 | 100 | | | | | | |
| 36 | male | 73 | CLL | 100 | 98.7 | | | | | | |
| 37 | male | 69 | CLL | 100 | 100 | | | | | | |
| 38 | male | 58 | CLL | 100 | 99.4 | | | | | | |
| 39 | male | 49 | CLL | 100 | 99 | | | | | | |
| 40 | male | 72 | CLL | 100 | 91.8 | | | | | | |
| 41 | female | 49 | CLL | 100 | 97.4 | | | | | | |
| 42 | female | 61 | CLL | 100 | 99 | 98 | 97.5 | 0.31 | | | 100 |
| 43 | male | 62 | CLL | 100 | 97.5 | 91.1 | 94 | 0 | | | 100 |
| 44 | male | 68 | CLL | 100 | 99.9 | 97.1 | 99.5 | 3.67 | | | 100 |
| 45 | female | 65 | CLL | 100 | 99.9 | 100 | 100 | 0 | | | 100 |
| 46 | male | 57 | CLL | 100 | 100 | 97 | 96.4 | 0 | | | 100 |
| 47 | male | 64 | CLL | 100 | 99.8 | 38.8 | 70.6 | 68.2 | | | 98.8 |
| 48 | male | 51 | CLL | 100 | 96.4 | 0.21 | 58.7 | 0.67 | | | 99.8 |

| | site density | | | | | | | |
|---|---|---|---|---|---|---|---|---|
| patient no. | CD32 | CD19 | CD20 | CD22 | CD23 | ROR1 | FcμR | CD32B |
| 1 | 43349 | 18155 | 3728 | 3465 | 3597 | 3878 | 4516 | |
| 2 | 50618 | 11426 | 3123 | 2937 | 6967 | 4388 | 7168 | |
| 3 | 28075 | 20581 | 24233 | 16446 | 41 | 11 | 4475 | |
| 4 | 29271 | 6348 | 870 | 1092 | 400 | 4295 | 1762 | |
| 5 | 40099 | 10000 | 1086 | 2341 | 120 | 4228 | 2624 | |
| 6 | 60984 | 38143 | 18922 | 27768 | 2133 | | | |
| 7 | 21833 | 19359 | 2250 | 8576 | 3630 | | | |
| 8 | 29775 | 20917 | 2779 | 9114 | 4463 | | | |
| 9 | 46353 | 21571 | 2577 | 6074 | 2562 | | | |
| 10 | 31439 | 18145 | 942 | 3611 | 1076 | | | |
| 11 | 36984 | 15099 | 1566 | 3691 | 1924 | 5250 | 5754 | |
| 12 | 63251 | 19148 | 51037 | 30012 | 897 | 112 | 8536 | |
| 13 | 33951 | 21065 | 3957 | 3764 | 2778 | 4438 | 6482 | |
| 14 | 26585 | 24203 | 44007 | 25007 | 0 | 2097 | 4150 | |
| 15 | 32045 | 23165 | 29322 | 8672 | 594 | 7009 | 7328 | |
| 16 | 48302 | 20528 | 6175 | 3527 | 6286 | 5535 | 6320 | |
| 17 | 50715 | 28140 | 4643 | 7249 | 2057 | 4955 | 6156 | |
| 18 | 32736 | 19670 | 8807 | 9272 | 8225 | 1941 | 4517 | |
| 19 | 42273 | 14021 | 4260 | 805 | 2203 | 5298 | 6182 | |
| 20 | 25342 | 10525 | 2028 | 2216 | 617 | 3944 | 2305 | |
| 21 | 22897 | 16210 | 3214 | 2347 | 3944 | 5369 | 4475 | |
| 22 | 50372 | 17970 | 21563 | 13699 | 344 | 963 | 3744 | |
| 23 | 39893 | 19953 | | | | 4239 | 10214 | |
| 24 | 66448 | 16667 | | | | 2731 | 6963 | |
| 25 | 25203 | 16816 | | | | 3090 | 6915 | |
| 26 | 57120 | 13762 | | | | 4584 | 3482 | |
| 27 | 36313 | 26893 | | | | 5678 | 8072 | |
| 28 | 32126 | 23201 | 6681 | | | | | |
| 29 | 23696 | 12302 | 1408 | | | | | |
| 30 | 56789 | 9930 | 24960 | | | | | |
| 31 | 25723 | 22776 | 3392 | | | | | |
| 32 | 29386 | 22801 | | | | | | |
| 33 | 40241 | 23428 | | | | | | |
| 34 | 23515 | 8926 | | | | | | |
| 35 | 31843 | 24630 | | | | | | |
| 36 | 38959 | 17722 | | | | | | |
| 37 | 66814 | 24880 | | | | | | |
| 38 | 57046 | 23669 | | | | | | |
| 39 | 55393 | 22990 | | | | | | |
| 40 | 54821 | 8577 | | | | | | |
| 41 | 44686 | 15263 | | | | | | |
| 42 | 36551 | 18466 | 22131 | 13289 | 239 | | | 31848 |
| 43 | 40439 | 14002 | 11764 | 14578 | 16 | | | 42497 |
| 44 | 45710 | 35179 | 49019 | 17262 | 486 | | | 52733 |
| 45 | 48335 | 23228 | 28253 | 35279 | 16 | | | 55137 |
| 46 | 51015 | 32777 | 17074 | 17310 | 1 | | | 48428 |
| 47 | 24438 | 16066 | 3689 | 6087 | 9161 | | | 23795 |
| 48 | 13677 | 8483 | 224 | 4169 | 296 | | | 16247 |

Markers' expression defined by flow cytometry using FMO and isotype controls. All these patients matched the diagnostic criteria for CLL according to NCCN clinical practice guidelines in oncology: Chronic Lymphocytic Leukemia/Small Lymphocytic Lymphoma, version 1.2020.
