# Supplementary material for: Homogeneously high expression of CD32b makes it a potential target for CAR-T therapy for chronic lymphocytic leukemia
Source: J Hematol Oncol. 2021 Sep 16;14:149. doi: 10.1186/s13045-021-01160-9 (PMC8447616; doi:10.1186/s13045-021-01160-9)
Supplement: Supplementary file 2 — Additional file 2. Materials and Methods. [file 13045_2021_1160_MOESM2_ESM.docx]

**Materials and Methods**

**Patient samples and cell lines**

CLL patient specimens were acquired from the Department of Clinical Laboratory of Institute of Hematology and Blood Diseases Hospital, Chinese Academy of Medical Sciences & Peking Union Medical College. Raji, MEC 1 and 293T cell lines were obtained from the Cell Resource Center of our institute. Raji cells were cultured in RPMI 1640 (Gibco, NY, USA) and 293T was cultured in DMEM (Hyclone, Logan, USA) media supplemented with 10% FCS. Raji cells for xenograft models were engineered to overexpress GFP-luciferase by lentiviral infection.

**CAR vector construction**

The anti-CD19-4-1BB-CD3ζ (CD19 CAR) or anti-CD32b-4-1BB-CD3ζ (CD32b CAR) [nucleotide sequence](https://www.baidu.com/link?url=sp3NyT1XKZu2b63DK2yPnvy8-HY5v1Iekabf2OIN9d6Pj9EB_wkYIfmrAXHIyYoutJlJPX6bhLIDYIhh5euwKJsxa4WHKU1eSVuDL0YtsZf6BV9ep0bjd4Bj98Q71faY&wd=&eqid=fa9345ea000dfa19000000035e0d7ecb), containing a single-chain variable fragment (scFv) from the murine anti-human CD19 antibody (clone FMC63), or the anti-human CD32b antibodies (clone 2B6 and clone NOV2108) [1, 2], which was fused to the 4-1BB and CD3-ζ intracellular domains as previously reported, were synthesized and inserted into pCDH-CMV-MCS-EF1-Puro plasmid.

**T cell isolation and manufacture of CAR-T cells**

CD32b and CD19 CAR lentivirus was packaged by transfecting 293T cells with packaging plasmids (REV, VSVG and PMDL) and pCDH that carried CD19 CAR or CD32b CAR. Culture supernatants were collected after 48 hours and precipitated by PEG-6000 overnight, and concentrated by centrifuge for 1 hour at 1500 *g*.

To generate CAR-T cells, T cells from healthy donors or patient peripheral blood were separated by RosetteSep™ Human T Cell Enrichment Cocktail (STEMCELL, Vancouver, CA) and stimulated with anti-CD3/CD28 antibody (STEMCELL), 0.1 μg/μl human IL-2, 0.01 μg/μl human IL-7, 0.01 μg/μl human IL-15, and 0.01 μg/μl human IL-21 (Peprotech, NJ, USA), and transduced with pCDH, CD32b CAR and CD19 CAR lentivirus.

**CAR detection by flow cytometry**

CD19 CAR expression was measured by a human CD19-Fc chimera protein (R&D Systems, Minneapolis, USA) and anti-human IgG Fc-APC antibody (BioLegend, San Diego, USA), and CD32b CAR expression was measured by a human FcγRIIB/C (CD32b/c)-His fusion protein (R&D Systems) and anti-His-APC Antibody (BioLegend).

**Cytotoxicity assay**

The cytotoxicity of CAR-T cells was evaluated by in vitro co-culturing with CD32^+^ Raji cells as previously reported. 2 × 10^4^ target cells were co-cultured with CAR-T cells in different ratio for 24 to 36 hours. We cultured the cells in 96-well round bottom plates (Corning, Lowell, USA) with triplicate wells/condition. CD19 or CD32 antibodies were stained for detecting target cells, and Precision Count Beads, DAPI and 7-AAD were added before detecting. DAPI^-^ 7-AAD^-^ represents the population of live cells. After staining, samples processed immediately on FACS Canto II, with each collection acquiring 1000 bead events. Specific lysis was calculated according to the residual live cell counts of experimental group and negative control group.

**Engineering anti-CD32b scFv-Flag antibodies**

[Nucleotide sequence](https://www.baidu.com/link?url=sp3NyT1XKZu2b63DK2yPnvy8-HY5v1Iekabf2OIN9d6Pj9EB_wkYIfmrAXHIyYoutJlJPX6bhLIDYIhh5euwKJsxa4WHKU1eSVuDL0YtsZf6BV9ep0bjd4Bj98Q71faY&wd=&eqid=fa9345ea000dfa19000000035e0d7ecb) of anti-CD32b scFv (clone 2B6 or NOV2108) fused to Flag was synthesized and inserted into pCDH-CMV-MCS-EF1-Puro vector to produce soluble anti-CD32b scFv-Flag antibodies by transfecting plasmids into 293T. Cell supernatants were acquired and centrifuged at 3000 rpm for 15 minutes. For verification of CD32b specificity and detection of CD32b expression, 2B6 scFv-Flag was incubated for 30 minutes at 4 °C with CD32a^+^ 293T cells, CD32b^+^ 293T cells or CD32a^-^ CD32b^-^ 293T cells, CLL cells, or PBMC cells (1-5×10^5^ cells/tube), which were subsequently analyzed through flow cytometry with a PE conjugated rat anti-Flag antibody.

**RNA sequencing**

Total RNA was sorted from CD5^+^CD19^+^ cells of CLL patients’ peripheral blood and extracted by TRIzol reagent (Invitrogen Life Technologies, Carlsbad, USA) and subjected to global transcriptome sequencing. Relative abundance of transcripts was estimated at both gene and transcript level (FPKM) by Novogene Genomics (Beijing, China).

**Flow cytometry**

Flow cytometry was undergone on Canto II or LSR II cell analyzer (both from BD Biosciences, San Jose, CA, USA) and analyzed by Flowjo (Tree Star). Cells for flow cytometry analysis were stained at 4 °C for 30 min in dark environment. Cells were resuspended in 200 μl PBS containing 2% FBS and 10 μl Precision Count Beads (BioLegend) (1×10^6^ /ml) were added to each sample if needed. Cell viability was determined by DAPI (Invitrogen) staining. We measured the antigens on CLL blasts, which was gated according to the expression of CD5, CD19, CD45 and other parameters, based on prior clinical diagnostic reports. Antibodies were purchased from BioLegend and BD Biosciences.

**Site density determination**

We used BD Bioscience QuantiBRITE PE beads to detect antigen site density on cell surface by measuring the antibody-binding capacity per cell (ABC). Cells stained by antibodies and precalibrated QuantiBRITE PE beads were measured with a FACSCanto II (BD Biosciences) under the same settings. The geometric mean of fluorescence of the antigen was acquired by the Flowjo software, comparing the known PE content of the QuantiBRITE beads to construct the standard curve. We then used QuantiBRITE standard curve to determine the ABC values under the measured geometric mean fluorescence.

The following antibodies were involved in flow cytometry analysis for site density determination：

anti-CD19-PE (clone SJ25C1), anti-CD32-PE (clone FUN-2), anti-FcμR-PE (clone 1E4), anti-ROR1-PE (clone 2A2), anti-CD20-PE (clone 2H7), anti-CD22-PE (clone S-HCL-1) and anti-CD23-PE (clone EBVCS-5) (BioLegend). Isotype control was mouse IgG1 κ isotype-PE (clone MOPC-21) or mouse IgG2b κ isotype-PE (clone MPC-11) (BioLegend).

**Gating strategies for human peripheral blood cells**

B cells were gated as CD19^+^, T lymphocytes were gated as CD3^+^, natural killer cells were gated as CD3^−^CD56^+^, dendritic cells were gated as HLA-DR^+^, CD11c^+^/CD123^+^, monocytes were gated as CD45^high^, SSC^dim^, CD33^+^, granulocytes were gated as CD45^dim^, SSC^high^, CD33^+^, platelets were gated as CD41a^+^, and erythrocytes were gated as CD45^−^CD235a^+^.

**Multiplex analysis of supernatants**

Cytokines in the culture supernatants were detected by “LEGENDplex Human CD8 Panel” immunoassay (BioLegend, San Diego, USA) after co-culturing of CAR-T cells with Raji or primary CLL cells. 25 μl supernatant was collected and added to the detection system. We used FACSCanto II cytofluorometer (Becton Dickinson) to record the data, and used the “LEGENDplex” Data Analysis software 8.0 to analyze data. The “LEGENDplex Human CD8 Panel” bead-based immunoassay can detect IL-2, IL-4, IL-6, IL-10, IL-17A, interferon-γ (IFN-γ), tumor necrosis factor-α (TNF-α), sFasL, perforin, granzyme A/B, and granulysin.

**Raji and CLL in vivo models**

The Raji model was built by injection of 3 × 10^5^ luciferase^+^ Raji cells through tail vein into 5-9 week-old NOD-*Prkdc^scid^* *Il2rg^tm1^*/Bcgen (NSG) mice (Biocytogen, Beijing, China) on day -5. Control T cells or 2B6bbz T cells (1 × 10^6^) were injected through tail vein on day 0. Mice were anesthetized and intraperitoneally injected with D-luciferin (YEASEN, Shanghai, China), then tumor burden in mice was measured by XenogenIVIS-200 Spectrum camera on day 0 to quantify engraftment and then weekly measured. Living Image Version 4.1 software (Caliper Life Sciences, Hopkinton, MA) was applied to take bioluminescent photograph and analyze the bioluminescent signals.

In CLL in vivo studies, NSG mice were irradiated in 150 cGy on day -3 and then CLL cells were injected in number of 2-4 × 10^7^ peripheral blood mononuclear cells (PBMCs) which contained more than 90% CLL blasts per mice. Three days after leukemia infusion, percentage of tumor cells in mouse PB (peripheral blood) were analyzed by flow cytometry to confirm successful engraftment, and mouse were divided evenly into three groups to ensure similar tumor burdens between different groups. Then 5 × 10^5^ CD32b CAR-T, CD19 CAR-T, or control T cells were infused into mice through tail vein. After tissue suspension was obtained from mice, T and tumor cells were detected by flow cytometry. Success engraftment of primary CLL leukemic cells was defined as ≥1% hCD32^+^hCD19^+^hCD5^+^ cells in the PB of NSG mice.

**Statistical analyses**

We conducted paired or unpaired two-tailed Student's t test, Pearson correlation analysis, Log-rank (Mantel-Cox) test or chi-square test in Prism (GraphPad, San Diego, USA). P value was calculated to indicate the statistical significance of differences. (It was considered significant if P values were below 0.05: **P < 0.05; **P < 0.01; ***P < 0.001.*)

**References:**

1. Veri MC, Gorlatov S, Li H, Burke S, Johnson S, Stavenhagen J, et al. Monoclonal antibodies capable of discriminating the human inhibitory Fcgamma-receptor IIB (CD32B) from the activating Fcgamma-receptor IIA (CD32A): biochemical, biological and functional characterization. Immunology. 2007, 121(3):392-404.

2. Balke N, Calzascia T, Ewert S, Harris A, Hute HA, Isnardi I, et al. ANTIBODIES TARGETING CD32b AND METHODS OF USE THEREOF. United States Patent Application Publication, 2017.
