# Supplementary material for: Homogeneously high expression of CD32b makes it a potential target for CAR-T therapy for chronic lymphocytic leukemia
Source: J Hematol Oncol. 2021 Sep 16;14:149. doi: 10.1186/s13045-021-01160-9 (PMC8447616; doi:10.1186/s13045-021-01160-9)
Supplement: Supplementary file 3 — Additional file 3: Fig. S1. CD32 expresses higher than other antigens on primary CLL samples. a Flow cytometric analysis of surface expression of CD32, CD19, CD20, CD22, ROR1, FcμR and CD23 in 4 CLL samples. CLL cells were gated as CD19+CD5+ cells. b Site density comparison between CD32 and CD19 in CLL patients (n = 41). c Quantification of mRNA transcripts from Raji cells, THP-1 cells and leukemic cells of 2 CLL patients by RNA sequencing. d 293T cells were genetically modified to express CD32a and CD32b; cells (unmodified 293T, CD32a+ 293T and CD32b+ 293T) were stained with 2B6-scFv-Flag Ab, 2108-scFv-Flag Ab and anti-CD32 mAb (clone FUN-2). Fig. S2. CD32b CAR-T against Raji cells in vitro and in vivo. a Flow cytometry analysis of CAR expression in T cells following lentiviral transduction. Left, control T cells; middle, T cells transduced with 2B6bbz; right, T cells transduced with 2108bbz. CARs were detected by CD32b-His followed by an anti-His-APC second antibody stain. b Flow cytometric analysis of surface expression of CD32b on the B-cell leukemia cell lines Mec-1 and Raji. c Antigen-specific cytokine production in response to CD32b+ Raji cells. 2B6bbz and control T cells were incubated with Raji cells (2 × 10^4^) respectively for 24 h in E: T ratio of 1:1. The various proteins in the culture supernatant were detected using the bead-based “LEGENDplex multi-analyte assay.” d Representative flow cytometric plot and flow gating strategy of peripheral blood from Raji-NSG mice 15 days after receiving 2B6bbz or control T cells. Fig. S3. CD32b CAR-T had potent cytotoxicity to primary CLL. a Flow cytometry analysis of CAR expression in T cells following lentiviral transduction. Left, control T cells; left-center, T cells transduced with 2B6bbz; right-center, T cells transduced with 2108bbz; right, T cells transduced with CD19 CAR. CARs were detected by CD32b-His/CD19-Fc followed by an anti-His-APC/anti-Fc second antibody stain. b Specific cytotoxicity of 2B6bbz, 2108bb [file 13045_2021_1160_MOESM3_ESM.pdf]

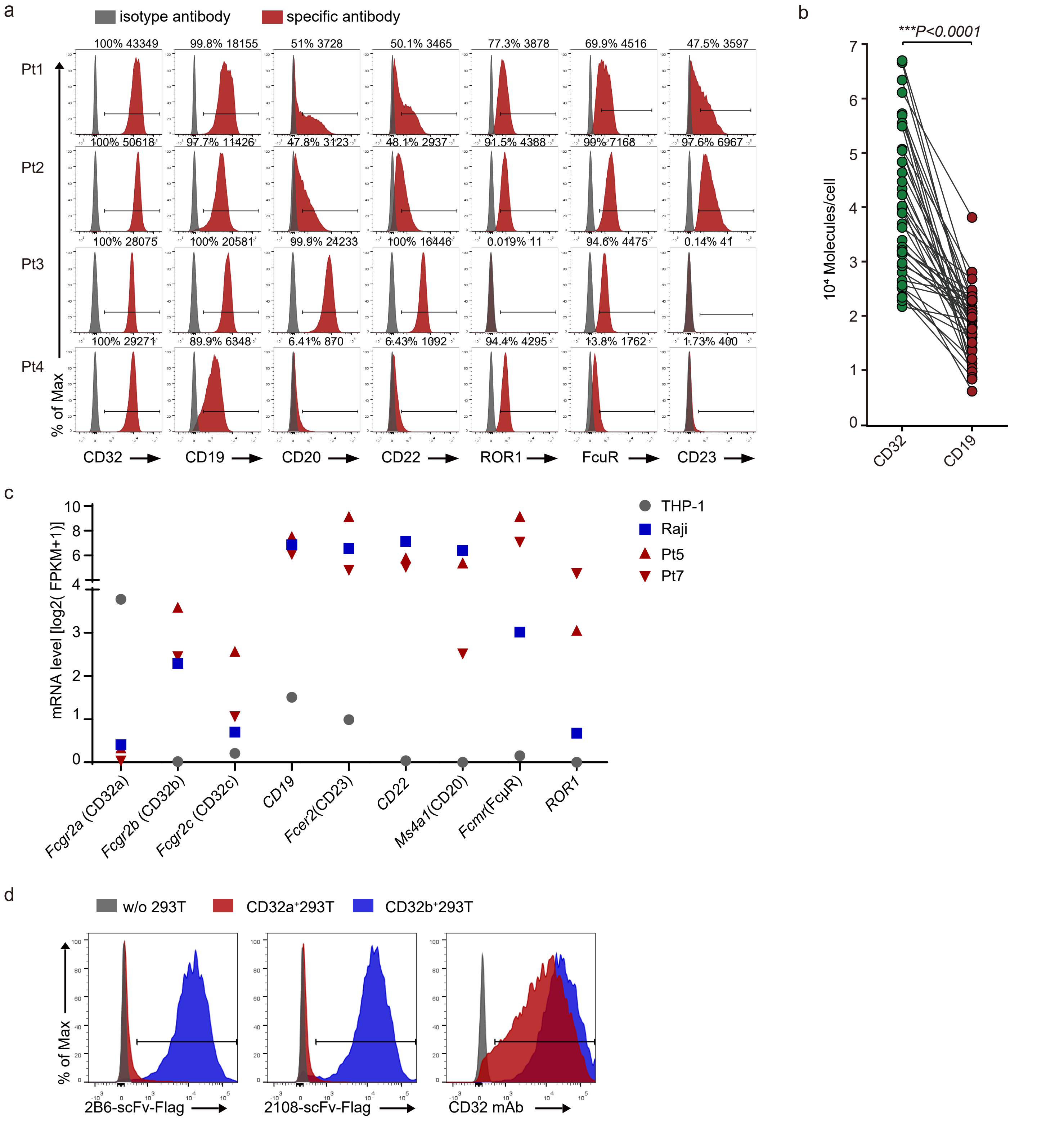
a
isotype antibody
specific antibody
Pt1
Pt2
Pt3
Pt4
% of Max
100% 43349
99.8% 18155
51% 3728
50.1% 3465
77.3% 3878
69.9% 4516
47.5% 3597
100% 50618
97.7% 11426
47.8% 3123
48.1% 2937
91.5% 4388
99% 7168
97.6% 6967
100% 28075
100% 20581
99.9% 24233
100% 16446
0.019% 11
94.6% 4475
0.14% 41
100% 29271
89.9% 6348
6.41% 870
6.43% 1092
94.4% 4295
13.8% 1762
1.73% 400
CD32
CD19
CD20
CD22
ROR1
FcuR
CD23
b
***P<0.0001
10⁴ Molecules/cell
CD32
CD19
c
mRNA level [log2(FPKM+1)]
THP-1
Raji
Pt5
Pt7
Fcgr2a (CD32a)
Fcgr2b (CD32b)
Fcgr2c (CD32c)
CD19
Fcer2(CD23)
CD22
Ms4a1(CD20)
Fcmr(FcμR)
ROR1
d
w/o 293T
CD32a⁺293T
CD32b⁺293T
% of Max
2B6-scFv-Flag
2108-scFv-Flag
CD32 mAb

Figure 1S

Fig. S1. CD32 expresses higher than other antigens on primary CLL samples. a Flow cytometric analysis of surface expression of CD32, CD19, CD20, CD22, ROR1, FcμR and CD23 in 4 CLL samples. CLL cells were gated as $CD19^{+}CD5^{+}$ cells. **b** Site density comparison between CD32 and CD19 in CLL patients (n=41). **c** Quantification of mRNA transcripts from Raji cells, THP-1 cells and leukemic cells of 2 CLL patients by RNA sequencing. **d** 293T cells were genetically modified to express CD32a and CD32b; cells (unmodified 293T, $CD32a^{+}$ 293T and $CD32b^{+}$ 293T) were stained with 2B6-scFv-Flag Ab, 2108-scFv-Flag Ab and anti-CD32 mAb (clone FUN-2). Paired two-tailed Student's t test was used in b (***$P < 0.001$).

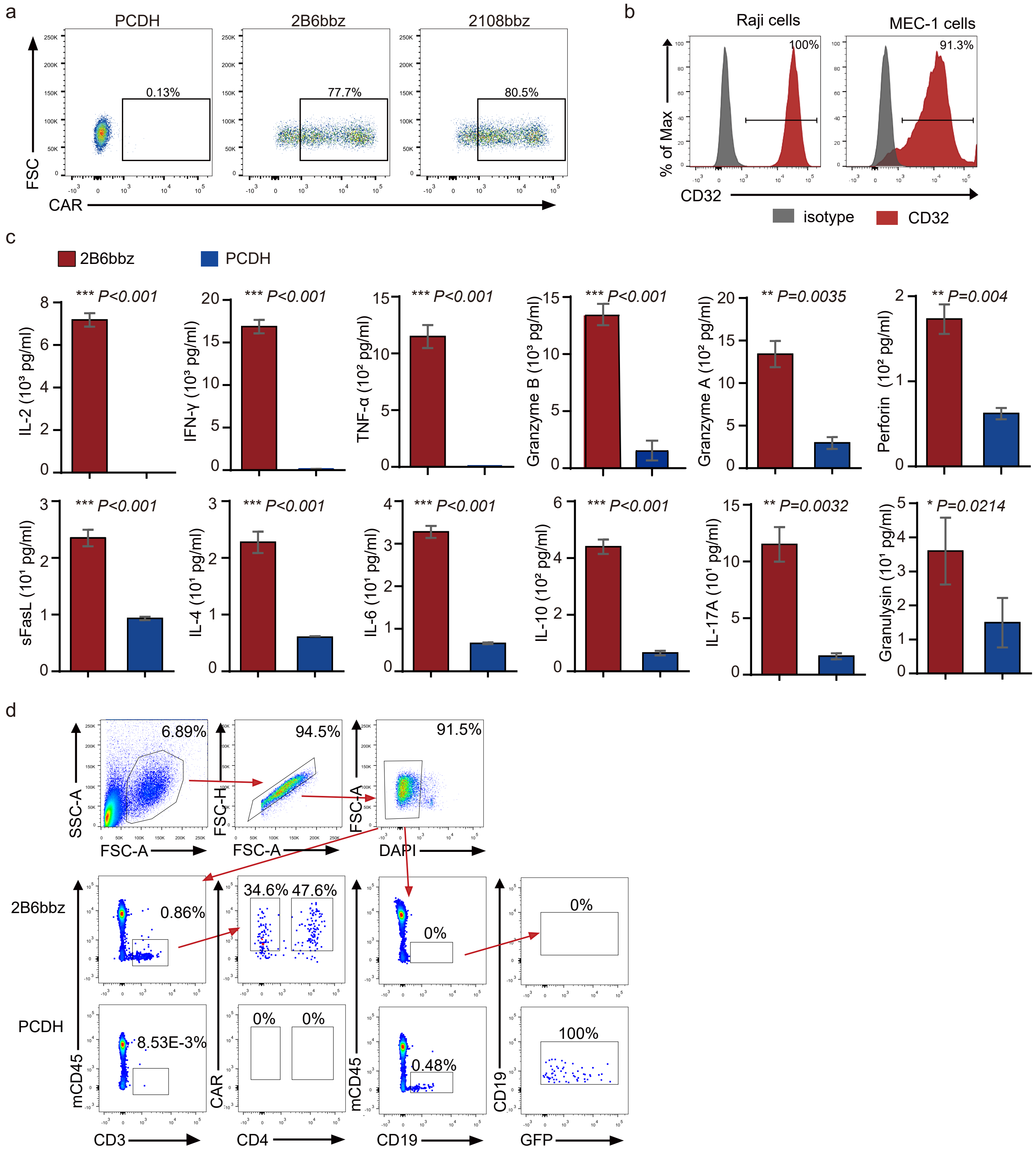
a
PCDH
2B6bbz
2108bbz
0.13%
77.7%
80.5%
FSC
CAR
b
Raji cells
MEC-1 cells
100%
91.3%
% of Max
CD32
isotype
CD32
c
2B6bbz
PCDH
*** P<0.001
** P=0.0035
** P=0.004
** P=0.0032
* P=0.0214
IL-2 (10³ pg/ml)
IFN-γ (10³ pg/ml)
TNF-α (10² pg/ml)
Granzyme B (10³ pg/ml)
Granzyme A (10² pg/ml)
Perforin (10² pg/ml)
sFasL (10¹ pg/ml)
IL-4 (10¹ pg/ml)
IL-6 (10¹ pg/ml)
IL-10 (10² pg/ml)
IL-17A (10¹ pg/ml)
Granulysin (10¹ pg/ml)
d
6.89%
94.5%
91.5%
SSC-A
FSC-A
FSC-H
DAPI
2B6bbz
PCDH
0.86%
34.6%
47.6%
0%
0%
8.53E-3%
0%
0%
0.48%
100%
mCD45
CAR
CD19
CD3
CD4
GFP

Figure 2S

Fig. S2. CD32b CAR-T against Raji cells *in vitro* and *in vivo*. a Flow cytometry analysis of CAR expression in T cells following lentiviral transduction. Left, control T cells; middle, T cells transduced with 2B6bbz; right, T cells transduced with 2108bbz. CARs were detected by CD32b-His followed by an anti-His-APC second antibody stain. **b** Flow cytometric analysis of surface expression of CD32b on the B-cell leukemia cell lines Mec-1 and Raji. **c** Antigen-specific cytokine production in response to $CD32b^{+}$ Raji cells. 2B6bbz and control T cells were incubated with Raji cells (2×10^4) respectively for 24 hours in E: T ratio of 1:1. The various proteins in the culture supernatant were detected using the bead-based "LEGENDplex multi-analyte assay." **d** Representative flow cytometric plot and flow gating strategy of peripheral blood from Raji-NSG mice 15 days after receiving 2B6bbz or control T cells. IFN-γ, interferon-γ; TNF-α, tumor necrosis factor α; IL-2, interleukin-2; IL-4, interleukin-4; IL-6, interleukin-6; IL-10, interleukin-10; IL-17A, interleukin-17A; sFasL, soluble Fas ligand. Unpaired two-tailed Student's t test was used for statistical analyses in c (*$P < 0.05$, **$P < 0.01$, ***$P < 0.001$).

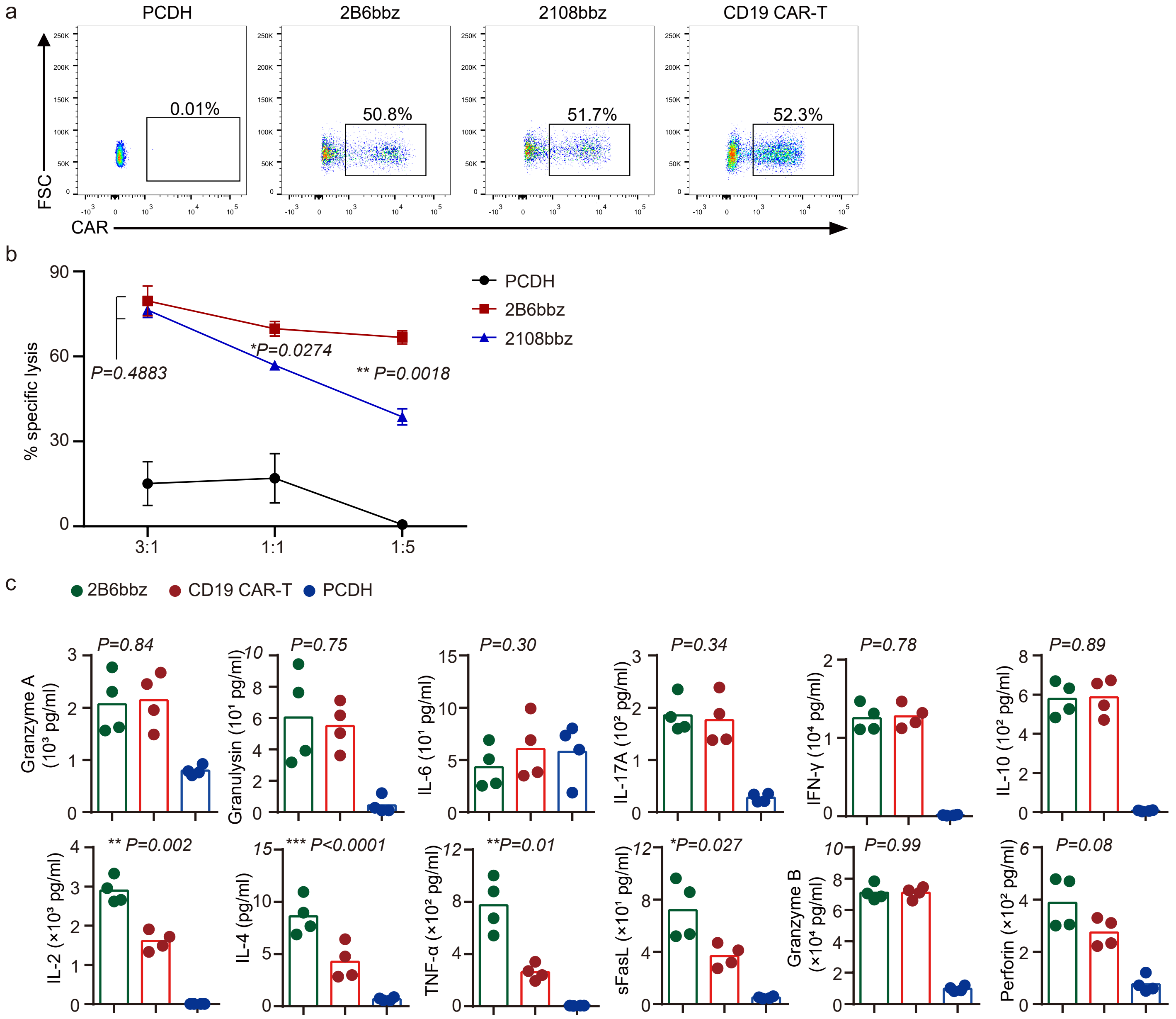
a
PCDH
2B6bbz
2108bbz
CD19 CAR-T
0.01%
50.8%
51.7%
52.3%
FSC
CAR
b
% specific lysis
PCDH
2B6bbz
2108bbz
P=0.4883
*P=0.0274
** P=0.0018
3:1
1:1
1:5
c
2B6bbz
CD19 CAR-T
PCDH
Granzyme A (10³ pg/ml)
P=0.84
Granulysin (10¹ pg/ml)
P=0.75
IL-6 (10¹ pg/ml)
P=0.30
IL-17A (10² pg/ml)
P=0.34
IFN-γ (10⁴ pg/ml)
P=0.78
IL-10 (10² pg/ml)
P=0.89
IL-2 (×10³ pg/ml)
** P=0.002
IL-4 (pg/ml)
*** P<0.0001
TNF-α (×10² pg/ml)
**P=0.01
sFasL (×10¹ pg/ml)
*P=0.027
Granzyme B (×10⁴ pg/ml)
P=0.99
Perforin (×10² pg/ml)
P=0.08

Figure 3S

Fig. S3 CD32b CAR-T had potent cytotoxicity to primary CLL. a Flow cytometry analysis of CAR expression in T cells following lentiviral transduction. Left, control T cells; left-center, T cells transduced with 2B6bbz; right-center, T cells transduced with 2108bbz; right, T cells transduced with CD19 CAR. CARs were detected by CD32b-His/CD19-Fc followed by an anti-His-APC/anti-Fc second antibody stain. **b** Specific cytotoxicity of 2B6bbz, 2108bbz or control T cells after coculture with primary CLL cells for 36 hours at the indicated E:T ratios; **c** Antigen-specific cytokine production of 2B6bbz, CD19 CAR-T and control T cells in response to 24 hours co-culturing with primary CLL cells. Unpaired two-tailed Student's t test was used for statistical analyses in b, c, (*$P < 0.05$, **$P < 0.01$, ***$P < 0.001$).

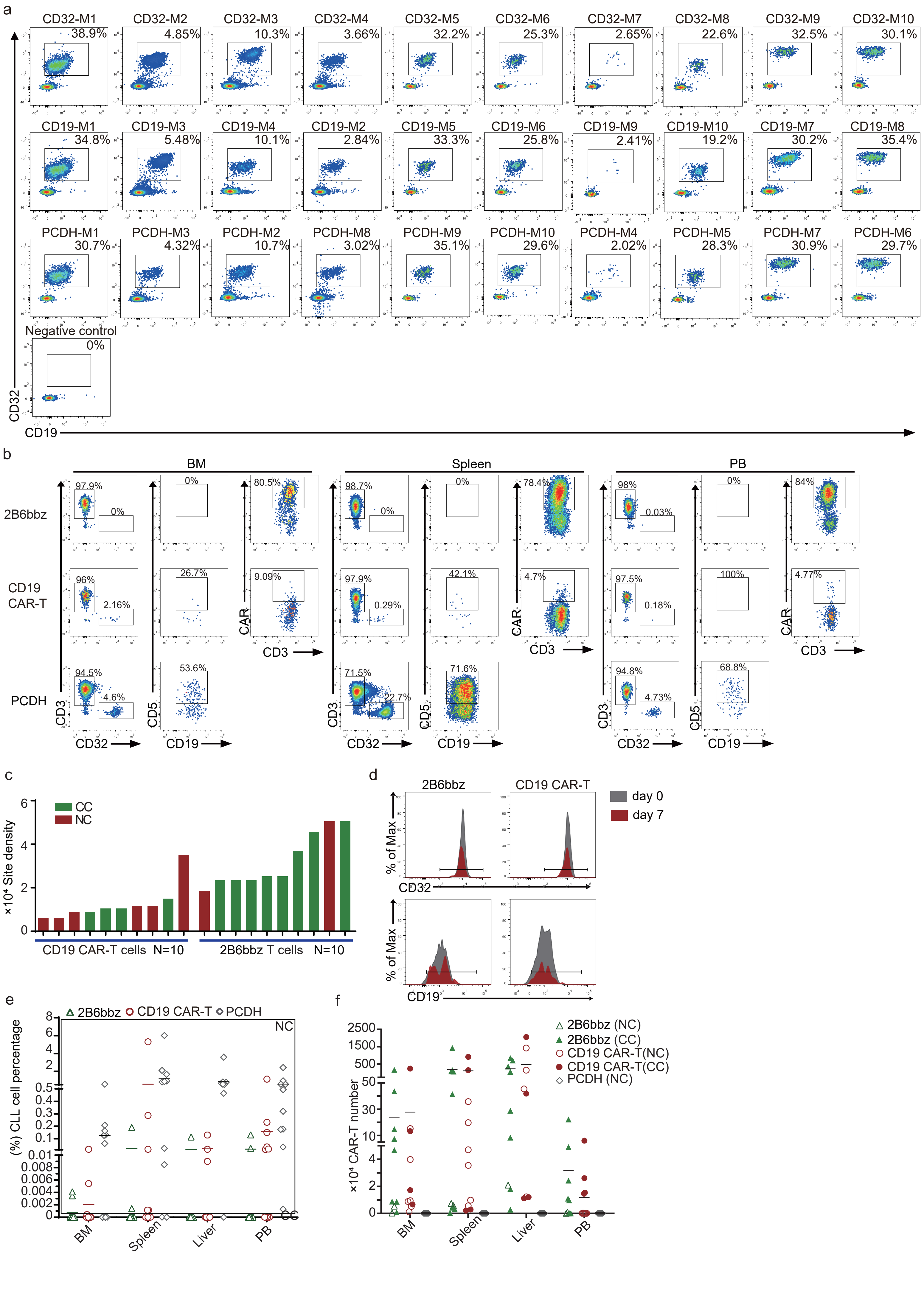
a
CD32-M1 38.9%
CD32-M2 4.85%
CD32-M3 10.3%
CD32-M4 3.66%
CD32-M5 32.2%
CD32-M6 25.3%
CD32-M7 2.65%
CD32-M8 22.6%
CD32-M9 32.5%
CD32-M10 30.1%
CD19-M1 34.8%
CD19-M3 5.48%
CD19-M4 10.1%
CD19-M2 2.84%
CD19-M5 33.3%
CD19-M6 25.8%
CD19-M9 2.41%
CD19-M10 19.2%
CD19-M7 30.2%
CD19-M8 35.4%
PCDH-M1 30.7%
PCDH-M3 4.32%
PCDH-M2 10.7%
PCDH-M8 3.02%
PCDH-M9 35.1%
PCDH-M10 29.6%
PCDH-M4 2.02%
PCDH-M5 28.3%
PCDH-M7 30.9%
PCDH-M6 29.7%
Negative control 0%
CD32
CD19
b
BM
Spleen
PB
2B6bbz
CD19 CAR-T
PCDH
CD3
CD32
CD5
CD19
CAR
c
CC
NC
×10⁴ Site density
CD19 CAR-T cells N=10
2B6bbz T cells N=10
d
2B6bbz
CD19 CAR-T
day 0
day 7
% of Max
CD32
CD19
e
2B6bbz
CD19 CAR-T
PCDH
NC
CC
(%) CLL cell percentage
BM
Spleen
Liver
PB
f
2B6bbz (NC)
2B6bbz (CC)
CD19 CAR-T(NC)
CD19 CAR-T(CC)
PCDH (NC)
×10⁴ CAR-T number
BM
Spleen
Liver
PB

Figure 4S

Fig. S4. 2B6bbz T against primary CLL cells *in vivo*. a Flow cytometric analysis of tumor percentage in peripheral blood before T cells infusion. **b** Representative flow cytometry plot of bone marrow, spleen and peripheral blood, from CLL-NSG mice after receiving CAR-T cells for 18 days. **c** Treatment response of 2B6bbz and CD19 CAR-T cells against mice transplanted with different antigen density primary CLL cells. **d** Flow cytometric analysis of CD32 and CD19 expression on CLL cells from peripheral blood of NSG mice before and at 7 days after CAR-T infusion. **e** Quantification of the percentage of CLL cells in peripheral blood, bone marrow, spleen and liver from CC and NC CLL-NSG mice after receiving CAR-T cells for 18 days. **f** Number of CAR-T cells in peripheral blood, bone marrow, spleen and liver from CC and NC CLL-NSG mice after receiving CAR-T cells for 18 days. CC, complete clearance (defined as tumor residual less than 0.001% in all the tissues detected); NC, not clearance (defined as a leukemia cell burden of greater than 0.001% in any tissue detected); BM, bone marrow; PB, peripheral blood.

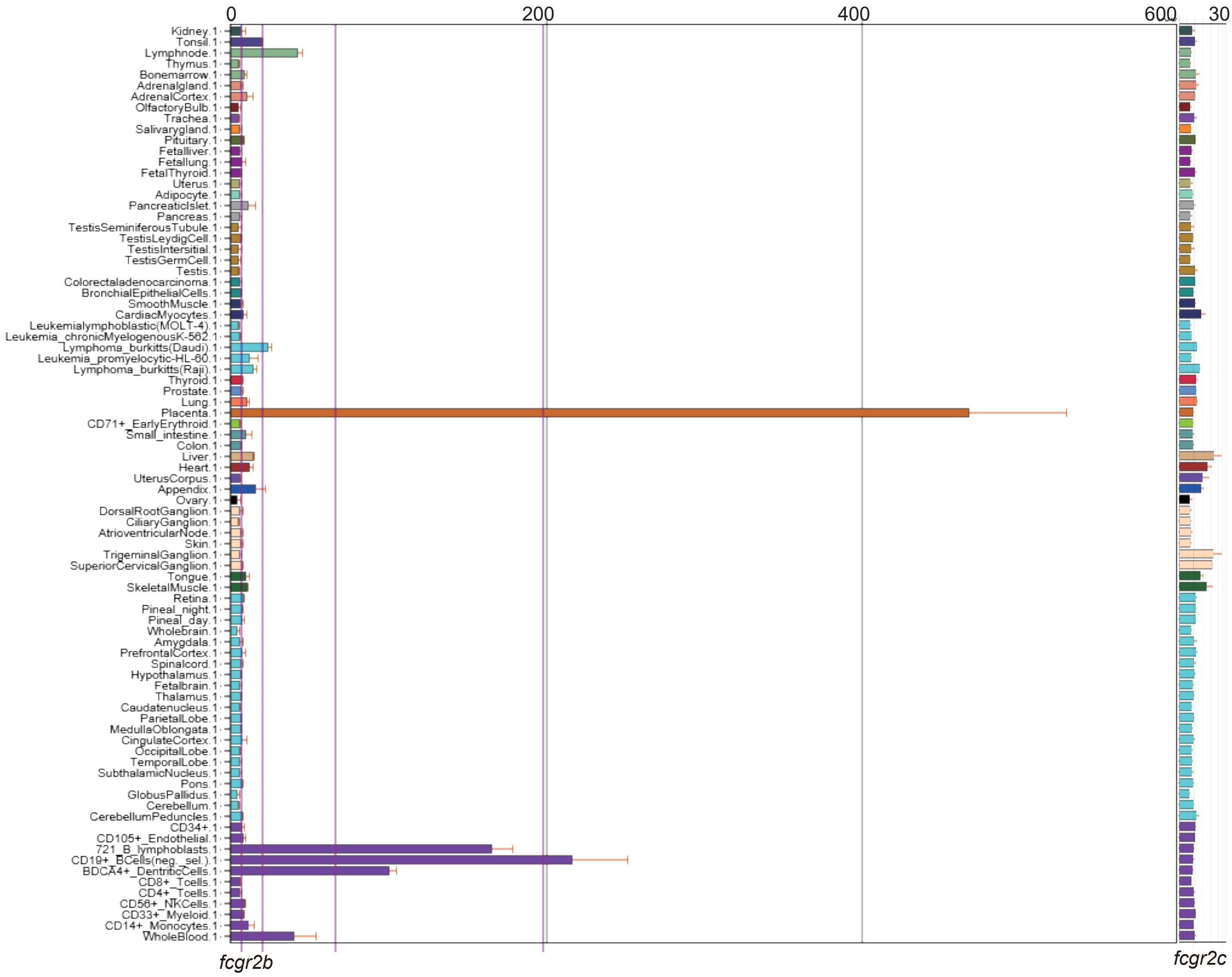
0
200
400
600
30
Kidney.1
Tonsil.1
Lymphnode.1
Thymus.1
Bonemarrow.1
Adrenalgland.1
AdrenalCortex.1
OlfactoryBulb.1
Trachea.1
Salivarygland.1
Pituitary.1
Fetalliver.1
Fetallung.1
FetalThyroid.1
Uterus.1
Adipocyte.1
PancreaticIslet.1
Pancreas.1
TestisSeminiferousTubule.1
TestisLeydigCell.1
TestisIntersitial.1
TestisGermCell.1
Testis.1
Colorectaladenocarcinoma.1
BronchialEpithelialCells.1
SmoothMuscle.1
CardiacMyocytes.1
Leukemialymphoblastic(MOLT-4).1
Leukemia_chronicMyelogenousK-562.1
Lymphoma_burkitts(Daudi).1
Leukemia_promyelocytic-HL-60.1
Lymphoma_burkitts(Raji).1
Thyroid.1
Prostate.1
Lung.1
Placenta.1
CD71+_EarlyErythroid.1
Small_intestine.1
Colon.1
Liver.1
Heart.1
UterusCorpus.1
Appendix.1
Ovary.1
DorsalRootGanglion.1
CiliaryGanglion.1
AtrioventricularNode.1
Skin.1
TrigeminalGanglion.1
SuperiorCervicalGanglion.1
Tongue.1
SkeletalMuscle.1
Retina.1
Pineal_night.1
Pineal_day.1
Wholebrain.1
Amygdala.1
PrefrontalCortex.1
Spinalcord.1
Hypothalamus.1
Fetalbrain.1
Thalamus.1
Caudatenucleus.1
ParietalLobe.1
MedullaOblongata.1
CingulateCortex.1
OccipitalLobe.1
TemporalLobe.1
SubthalamicNucleus.1
Pons.1
GlobusPallidus.1
Cerebellum.1
CerebellumPeduncles.1
CD34+.1
CD105+_Endothelial.1
721_B_lymphoblasts.1
CD19+_BCells(neg._sel.).1
BDCA4+_DentriticCells.1
CD8+_Tcells.1
CD4+_Tcells.1
CD56+_NKCells.1
CD33+_Myeloid.1
CD14+_Monocytes.1
WholeBlood.1
fcgr2b
fcgr2c

Figure 5S

Fig S5 mRNA expression profile of *fcgr2b* and *fcgr2c* in normal human tissues at mRNA level according to publicly available database (BioGPS: *fcgr2b*, http://biogps.org/#goto= genereport&id=2213; *fcgr2c*, http://biogps.org/#goto=genereport &id =9103;)
